# Age-Dependent Dissimilarity of the Nasopharyngeal and Middle Ear Microbiota in Children With Acute Otitis Media

**DOI:** 10.3389/fgene.2019.00555

**Published:** 2019-06-19

**Authors:** Silvio D. Brugger, Julia G. Kraemer, Weihong Qi, Lindsey Bomar, Anne Oppliger, Markus Hilty

**Affiliations:** ^1^Institute for Infectious Diseases, Faculty of Medicine, University of Bern, Bern, Switzerland; ^2^Department of Oral Medicine, Infection and Immunity, Harvard School of Dental Medicine, Boston, MA, United States; ^3^Department of Infectious Diseases and Hospital Epidemiology, University Hospital Zurich – University of Zurich, Zurich, Switzerland; ^4^Institute for Work and Health, University of Lausanne, University of Geneva, Épalinges, Switzerland; ^5^Functional Genomics Center Zurich, Swiss Federal Institute of Technology Zurich, University of Zurich, Zurich, Switzerland; ^6^Department of Microbiology, The Forsyth Institute, Cambridge, MA, United States

**Keywords:** nasopharyngeal microbiota, bacterial families, toddlers, acute otitis media, middle ear fluid, age

## Abstract

Acute bacterial otitis media is usually caused by otopathogens ascending to the middle ear from the nasopharynx (NP). However, it is unknown if the nasopharyngeal microbiota of children with acute otitis media (AOM) can serve as an age-dependent or independent proxy for the microbial communities of the middle ear fluid (MEF) as there is a lack of 16S rRNA amplicon sequencing studies simultaneously analyzing the microbial communities of the two sites. Within this study, we performed 16S rRNA next generation sequencing on a total of 286 nasopharyngeal swabs (NPSs) collected between 2004 and 2013 within a Swiss national AOM surveillance program from children (0–6 years) with AOM. In addition, 42/286 children had spontaneous tympanic membrane perforation and, therefore, those MEF could also be analyzed. We found that alpha [Richness, Shannon diversity index (SDI) and Evenness] and beta diversity measurements of the nasopharyngeal bacterial microbiota showed a clear dependency of the increasing age of the children. In more detail, bacterial richness and personalized profiles (measured by beta dispersion) were higher and more frequent in older children, respectively. Dissimilarity values based on the binary distance matrix of the microbiota patterns of the NP and the MEF also correlated with increasing age. In general, positive (PPV) and negative predictive values (NPV) of the most abundant operational taxonomic units (OTUs) in the NP were moderately and well predictive for their presence in the MEF, respectively. This data is crucial to better understand polymicrobial infections and therefore AOM pathogenesis.

## Introduction

Acute otitis media (AOM) is one of the most frequent pediatric diseases with a peak incidence at 6–12 months of age and is still responsible for high rates of antibiotic prescription (Lieberthal et al., 2013). Conventionally, bacterial or viral culturing of the MEF has been used as the gold standard for establishing microbial etiology of AOM (Yatsyshina et al., 2016). Traditionally, *Streptococcus pneumoniae*, non-typeable *Haemophilus influenzae* and *Moraxella catarrhalis* have been reported as the most common bacterial causes of AOM (Post et al., 1995; Kilpi et al., 2001; van Dongen et al., 2013; Kaur et al., 2014, 2017). However, the incidences of the pathogens causing AOM seem to be age-dependent, e.g., it has been reported that the incidence of pneumococcal AOM peaks around 12 months of age, whereas the incidence of *M. catarrhalis* AOM first peaks at 6 months and *H. influenzae* AOM at 20 months (Kilpi et al., 2001; Kaur et al., 2014).

A clinically important question to address, is whether sampling the NP provides an accurate estimate of the presence of the various microorganisms involved in otitis media as access to the NP is much less invasive than that to the middle ear (tympanocentesis) (van Dongen et al., 2013). Furthermore, it is hypothesized that bacterial AOM is caused by otopathogens residing in the nasal passages by ascension through the Eustachian tube under disturbance of the normal homeostasis (Coticchia et al., 2013). It has been described that nasopharyngeal colonization with the bacterial species most known to cause AOM is associated with middle ear disease with odds ratios (ORs) of approximately 2 (Faden et al., 1997; Revai et al., 2008). However, all of these studies are culture based or used specific PCR protocols targeting distinct species (Yatsyshina et al., 2016). There is a lack of 16S rRNA next generation sequencing studies characterizing the whole bacterial microbiota, rather than single species, with paired samples of NPSs and MEF of patients with AOM as well as of studies investigating the nasopharyngeal microbiota in toddlers and prepubertal children. Some very recent studies by us and others analyzing either the middle ear (Sillanpaa et al., 2017) and/or the NP (Hilty et al., 2012; Chonmaitree et al., 2017; Lappan et al., 2018) have already shown that a broader, sequencing-based survey of the bacterial microbiota provides additional important information which cannot be derived by bacterial cultures. Therefore, the overarching aim of this study was to characterize the bacterial, nasopharyngeal microbiota in Swiss children with AOM and to simultaneously characterize the bacterial microbiota of MEF and NPS in patients with AOM who also had a perforated tympanic membrane, at different ages. In more detail we aimed at (a) characterizing the nasopharyngeal microbiota of children with AOM aged <7 years and at (b) simultaneously characterizing the microbiota of NP and MEF of 42 children with spontaneous tympanic membrane perforation in different age groups in order to predict if and to what extend the microbiota of NPS may serve as proxy for the microbiota of MEF.

## Materials and Methods

### Study Design

This study included outpatients with AOM between 2004 and 2013. All children aged less than 7 years were included. Children were observed within a Swiss nationwide prospective surveillance system for various diseases, which is ongoing since 1998 and has been explained previously (Muhlemann et al., 2003; Allemann et al., 2017). The diagnosis of AOM episodes was done according to Centers for Disease Control and Prevention definitions which included two or more of the following symptoms: fever (>38°C), inflamed or painful or retracted tympanic membrane, reduced mobility of the tympanic membrane or fluid behind the tympanic membrane on otoscopy. Patients were eligible to be signed up repeatedly for each new episode of AOM (Muhlemann et al., 2003; Allemann et al., 2017). Participating physicians seeing AOM patients sampled the NP and provided a sample of the MEF in case of spontaneous rupture of the tympanic membrane. In total, 42/286 of participants provided both, MEF and a NPS. Tympanocentesis for routine surveillance was not intended in the surveillance system and was fully up to the decision of the treating physician. Participating physicians were only asked to swab MEF of spontaneously ruptured tympanic membranes. Nasopharyngeal samples analysis included the data from a previous study for children age 0–1 years (Hilty et al., 2012). NPS samples from the other age groups (>1 year but <7 years) and MEF samples are presented for the first time in this study. The overall AOM surveillance program is part of the governmental public health surveillance and is therefore exempted from approval by Institutional Review Boards. Informed consent was received from the parents.

### Sample Collection

Nasopharyngeal swabs were obtained through the nose reaching the NP using twisted wire rayon tipped applicators (Copan Ventury Transystem; Copan, Italy), and MEF were gained using the identical or cotton tip swabs (Allemann et al., 2017). Concerning the latter, the amount of time between spontaneous perforation and collection of samples was not recorded. With each sample, practitioners submitted a standardized questionnaire comprising the following patient data: age, gender, vaccination status, preceding antibiotic therapy, day care attendance, AOM history (number of otitis media episodes within the last 12 months) and place of residence. Culture and subsequent serotyping for *S. pneumoniae* was done for all NPS and MEF as described (Allemann et al., 2017).

### PCR Amplification and Amplicon Sequencing of 16S rRNA Genes

Amplification by PCR and amplicon sequencing has been described elsewhere (Hilty et al., 2012). In brief, DNA was extracted using 200 μl of transport medium, followed by amplification of bacterial 16S rRNA variable regions V3-V5 by MID tagged primer pair 341F/907R (*Escherichia coli* numbering). The final primer sequences including MID and adaptor sequences were A-341F, 5′-cgtatcgcctccctcgcgccatcag-[NNNNNNNNNN]-*ACTCCTACGGGAGGCAGCAG*-3′ and B-926R, 5′-ctatgcgccttgccagcccgctcag-[NNNNNNNNNN]-*CCGTCAATTCMTTTGAGTTT-*3′. PCR reactions were eluted by 40 μl double distilled water and the eluate was measured by Agilent 2100 Bioanalyzer (Agilent Technologies, Basel, Switzerland). PCR products with a concentration <1.0 ng/μl were excluded from the study. This corresponds to <1 pg/μl bacterial DNA as evaluated by quantitative PCR of pneumococcal DNA at different dilutions (Mika et al., 2015). A minimum of 1 pg/μl of bacterial DNA has been suggested as the cut-off when dealing with low density sample material (Biesbroek et al., 2012). Resulting PCR products were evenly pooled (40 ng), whereas every MID was used once, resulting in eight amplicon pools (Hilty et al., 2012). The amplicon pools were sequenced using the 454 sequencing platform. The resulting reads were submitted to the National Center for Biotechnology Information Sequence Read Archive (accession numbers SRR077426, SRR086165, SRR086166, and PRJEB23228).

### Analysis of Sequence Reads

Several quality control steps were performed for the “raw” 454 sequence reads to circumvent artificial inflation of diversity estimates (Kunin et al., 2010). First, forward reads were trimmed to 230 base pairs (bp) with shorter reads being rejected. The length of 230 bp was selected because the quality scores generally dropped for bases after this cut off as we have already previously shown (Hilty et al., 2012). After the additional trimming of MID and primer sequences, the actual sequences subjected to microbial diversity analysis were 200 bp long. It has been previously hypothesized that the 200 bp long forward reads are appropriate for accurate microbial community analysis (Liu et al., 2007). Second, reads having any unresolved bases (“N”s) or homopolymers longer than 8 bp were excluded, as well as singleton reads (reads, which appear only once in all samples and are therefore likely to be the product of polymerase errors). Finally, trimmed reads were filtered with a modified version of the quality filtering script in Pyrotagger (Kunin and Hugenholtz, 2010). The script removes first reads containing sequence error(s) in its MID and/or primer, and then discards reads in which over 15% of bases show a quality score lower than 20.

### Taxonomic Assignments and Calculation of Relative Bacterial Abundances of Operational Taxonomic Units (OTUs) and Bacterial Families

Pyrotagger was also used for the definition of OTUs, estimation of chimeras, and taxonomy assignments as previously described (Hilty et al., 2012). In brief, reads were clustered at a 97% sequence identity threshold using the Pyroclust algorithm. Putative chimera clusters flagged by Pyrotagger were discarded. The most abundant unique sequence was then chosen as a cluster representative and classified by comparison to Phylodb, which contains reference sequences exported from Greengene and SILVA databases (Kunin and Hugenholtz, 2010). An in house perl script was additionally used to collate family level taxonomic abundance for each sample by summing the number of reads belonging to a given family.

Within this study, the five most abundant families were analyzed separately, whereas all the remaining families were grouped as “others.” These analyses included the calculations of mean values and SEM (standard error of the mean) of the relative abundances per age group (0–1, 1–2, 2–3, 3–4, 4–5, 5–6, and 6–7 years).

### Alpha Diversity Calculations of NPS

Alpha diversity [Richness (S), SDI, and Evenness], referred to as within-community diversity (Lemon et al., 2012), was assessed for OTUs based on 97% sequencing identity.

Richness is defined by the total number of OTUs while evenness refers to the similarity in OTU relative abundance in a bacterial community. Shannon’s index accounts for both abundance and evenness of the species present. Values were calculated in R^1^, using the respective function of the *vegan* package. SDI and Richness values were log-transformed to achieve normally distributed residuals. Values of log-transformed Richness (S), log-transformed SDI, and Evenness were grouped according to the age of the study participant and regression analysis was performed. Shannon or Shannon–Weaver (or Shannon–Wiener) index is defined as *H = -sum p_i log(b) p_i*, where *p_i* is the proportional abundance of species *i* and *b* is the base of the logarithm.

Evenness index is defined as J′ = H′/H′_max_, where H′ is the number derived from the SDI and H′_max_ is the maximum possible value of H′. To correct for the dependency nature of the study, a linear mixed model including fixed effects [different age groups, the year of sampling, previous antibiotic treatment of the patient (yes or no), region of origin (West or East of Switzerland) and day care attendance (yes or no)] was done using the package lme4 in R (lm command). SDI and Richness values were log-transformed to achieve normally distributed residuals and outcomes were visualized according to the different age groups [e.g., for SDI: model.sdilog < -lm(df.SDI$SDIlog∼df.SDI$age + df.SDI$year + df.SDI$antibiotic + df.SDI$Region + df.SDI$day_care, na.action = na.exclude)].

### Beta Diversity Analyses of NPS

Beta-diversity (between-sample diversity) was measured by the weighted Ružička index (abundance-based) and the unweighted Jaccard index (presence/absence-based) of dissimilarity. Jaccard index is computed as 2B/(1+B), where B is Bray–Curtis *(d[jk] = (sum abs(x[ij]-x[ik])/(sum (x[ij]+x[ik])))* dissimilarity using the binary = TRUE flag. Ružička index is calculated identical to the Jaccard index but binary = FALSE. Pairwise distances between samples were received by the *vegdist* function and the resulting matrices were included to create NMDS plots (*metaMDS* function). Significant groupings between samples were assessed by a permutational multivariate analysis of variance using 1000 Monte Carlo permutation tests (PERMANOVA; *adonis* function). The multivariate dispersion of each sample group was determined by calculating the average distance (based on Jaccard and Ružička indices) to the sample type’s centroid using the *betadisper* function, and significant differences were assessed with Tukey’s Honest Significant Difference Test (TukeyHSD function). Boxplots and NMDS plots were generated in R utilizing the ggplot2 package.

### Analyses of Paired Samples (NPS and MEF)

Heat maps of the most abundant OTUs were plotted using the *ComplexHeatmap* function in R. Dissimilarity values between paired NP and MEF samples were derived by the weighted Ružička (abundance-based) and the unweighted Jaccard (presence/absence-based) distance matrices. Sensitivity/specificity and PPV/NPV including 95% CIs for the most abundant OTUs and pneumococcal serotypes were calculated. Linear correlation of weighted (Ružička) or unweighted (Jaccard) paired dissimilarity values and age (in months) of the patients with AOM were calculated using Graph pad prism.

## Results

### Study Population and Sample Processing

A total of 286 NPS from children (<7 years) with AOM were collected from 2004–2013. Samples were roughly equally distributed for sex and region of origin (i.e., west as compared to east). Very young children (0–3 years of age) were more likely to attend a day care and were more likely to be vaccinated with either PCV7 or PCV13. Detailed information on the study population, including previous antibiotic exposure and previous otitis media episodes, is listed in Table 1. In addition, 42/286 children were reported having tympanic membrane perforation and, therefore, provided both, MEF and a NPS. The microbiota of all samples was analyzed. After exclusion of low-quality reads, this resulted in 299’966 (mean, 1048.8; SD), ±940) and 39’726 (mean, 945.9; SD, ±580.9) high quality sequence reads for the NPS and MEF samples, respectively. Comparing the values for extrapolated true richness and extrapolated estimated richness indices revealed a coverage of 66.8 and 65.4% for the NPS (Supplementary Figure 1) and MEF (Supplementary Figure 2), respectively.

**Table 1:** Overview of study population/samples.

		Age of patients with episodes of AOM						
		0–1 years	1–2 years	2–3 years	3–4 years	4–5 years	5–6 years	6–7 years
Total number of NPS		65	108	8	36	33	21	15
**Year**								
	2004–2006	30	27	2	11	14	11	9
	2007–2009	30	76	3	20	16	8	5
	2010–2012	5	5	3	5	3	2	1
**Day care visits**							
	Yes	24	40	3	18	11	4	1
	No	40	59	5	18	21	16	13
	NA							
**Region**								
	East	25	41	1	18	23	9	7
	West	39	67	7	18	8	12	8
	NA							
**Gender**								
	Female	35	51	4	17	10	10	6
	Male	30	56	4	19	23	11	9
	NA	0	1	0	0	0	0	0
**Vaccination status**							
PCV7/PCV13	yes	31	68	5	14	4	1	0
	no	30	35	3	21	29	20	14
	NA	4	5	0	1	0	0	1
**Antibiotics during the last 8 weeks**							
	No	42	74	5	34	31	21	15
	Yes	15	26	3	2	1	0	0
	NA	8	8	0	0	1	0	0
**AOM history**								
	No	36	49	0	20	23	16	9
	≥1 previous episode	26	50	7	14	7	1	2
	NA	3	7	1	2	3	4	4


*AOM, acute otitis media; NPS, nasopharyngeal swabs; PCV, pneumococcal conjugate vaccine.*

### Dynamics of Bacterial Families Within NPS

**FIGURE 1:**
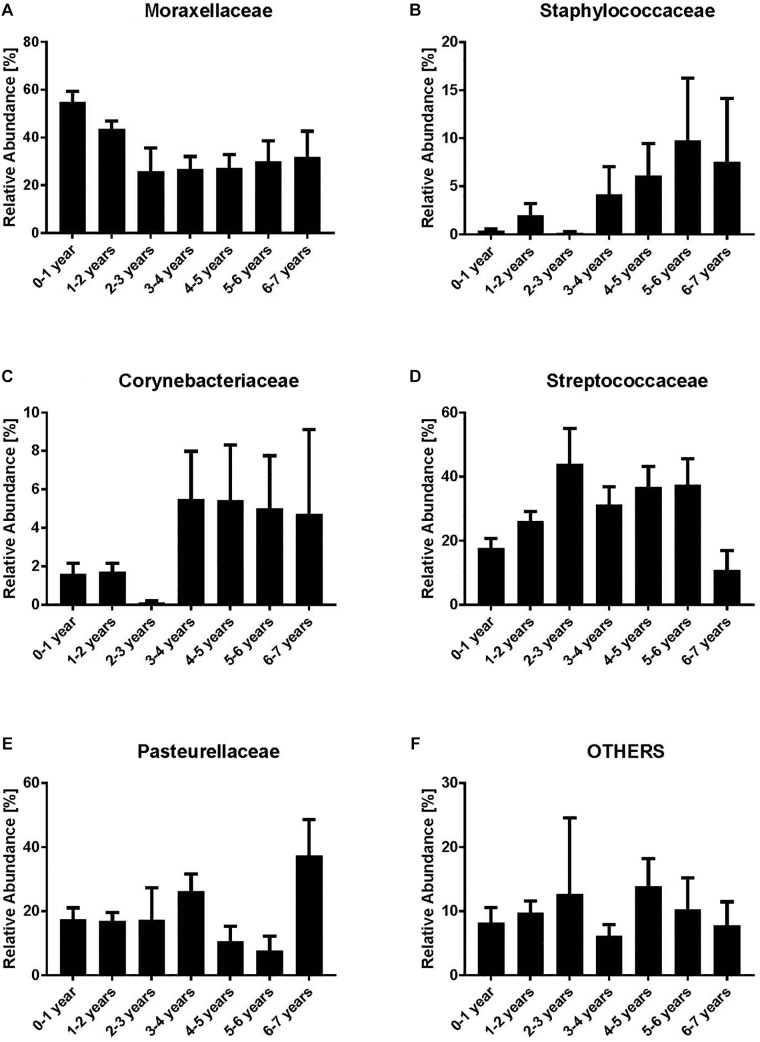
Relative abundances of bacterial families [Moraxellaceae **(A)**, Staphylococcaceae **(B)**, Corynebacteriaceae **(C)**, Streptococcaceae **(D)**, Pasteurellaceae **(E)** and Others **(F)**]. Indicated are the most abundant bacterial families while all the remaining are grouped as “others.” Bars represent mean values according to the age of the children with acute otitis media on the *x*-axis (in years). Moraxellaceae is the most abundant family within the younger age groups, whereas in older age groups Corynebacteriaceae and Staphylococcaceae are most abundant.

We analyzed the dynamics of the five most abundant bacterial families (Moraxellaceae, Streptococcaceae, Corynebacteriaceae, Pasteurellaceae, and Staphylococcaceae) and all remaining families (“others”). Moraxellaceae was the most abundant bacterial family with a maximum relative abundance within the first year of life (mean 54.7%) (Figure 1A). However, a clear decrease of Moraxellaceae from the 3rd to the 6th year of life was noted. In contrast, we observed an increase of Staphylococcaceae and Corynebacteriaceae with increasing age (Figure 1B,C). No clear monotonic changes were noted for Streptococcaceae, Pasteurellaceae and others (Figure 1D–F) though the relative abundance of Staphylococcaceae and Corynebacteriacecae decreased at age 2–3 and increased between 3–4 years, and, the relative abundance of Streptococcaceae slightly decreased between 3–4 years. Due to the low number of NP samples at age 2–3 (*n* = 8), the observed non-monotonic changes might be coincidental.

### Bacterial Richness of the Nasopharyngeal Microbiota Increases With Age in Children With AOM

**FIGURE 2:**
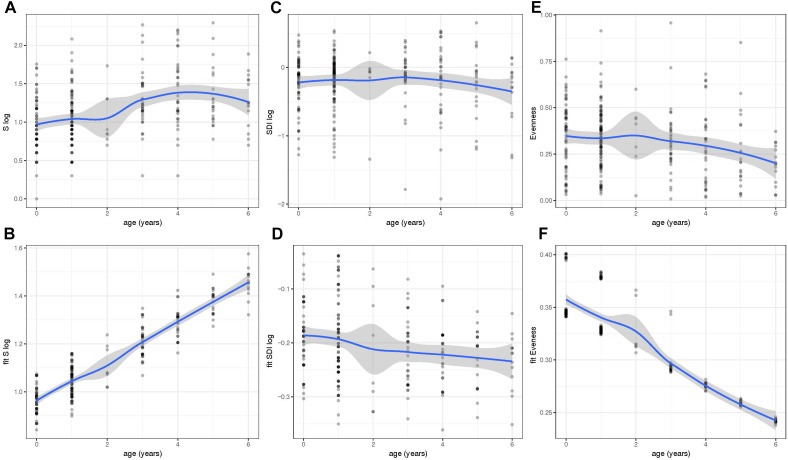
Within-community diversity [Richness (S), Shannon Diversity Index (SDI) and Evenness] analysis in different age groups. Values of S **(A)**, SDI **(C)**, and Evenness **(E)** were obtained at different ages (0–6 years of age). However, the residuals of the models for S and SDI were not normally distributed and, therefore, were log transformed **(A–D)**. To correct for the dependency nature of the study, linear mixed models including fixed effects were also done and fitted values of S **(B)**, SDI **(D)**, and evenness **(F)** were plotted throughout time. Richness and Evenness values reveal an increase and decrease in older children, respectively. Fitted values were obtained using the lme4 R package and plots were generated using ggplot2.

We next determined the values for the alpha diversity indices of richness (S), SDI, and evenness based on 97% OTUs (Figure 2A–F). The residuals of the models for S and SDI were not normally distributed and, therefore, were log transformed (Figure 2A–D). To correct for the dependency nature of the study, linear mixed models including fixed effects were done (Figure 2B,D,F). Results showed that the bacterial richness (S) based on OTUs with 97% sequence identity, increased with age and was highest in children with 6 years of age (Figure 2A,B). Increase was significant using a linear mixed effect (LM) model with fixed effects age, year of sample collection, previous antibiotic usage, day care attendance and geographical origin (Figure 2B) (*P* < 0.001). In contrast, there was no such trend for SDI (Figure 2C,D), whereas for evenness, we observed a significant decrease between younger and older age groups (*P* < 0.001) and values were lowest at 6 years of age (Figure 2E,F).

### Beta Diversity Analyses Revealed Clustering of NPS According to Age

**FIGURE 3:**
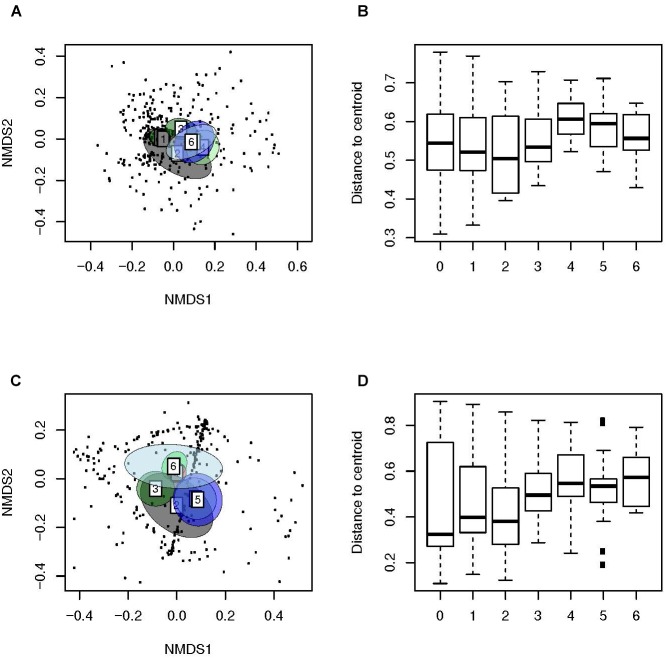
Beta-diversity analyses of samples of children with AOM suggest a more personalized bacterial microbiota with age. Illustrated are **(A)** Weighted (Ružička) and **(C)** unweighted (Jaccard) distances in microbiota composition, reduced in a 2D-space by using NMDS, 95% confidence ellipse for the group centroid shown. In addition, shown are the beta-dispersion based on **(B)** Ružička and **(D)** Jaccard dissimilarity indices in each sample type. The boxplots represent median (midline), interquartile ranges (shaded boxes), and ranges (whiskers). Data is shown according to the age (years) of the children with AOM.

We then used weighted (Figure 3A,B) and unweighted distance matrices (Figure 3C,D) to create ordination method based NMDS (Figure 3A,C) and multivariate dispersion plots (Figure 3B,D) for the analysis of Beta diversity. The ordination method based NMDS plots (Figure 3A,C) showed a distinct clustering of the microbiota of the NP of children with AOM according to age (PERMANOVA, unweighted: *F*-value: 6.6, *P* < 0.01, weighted: *F*-value: 6.3, *P* < 0.01). This indicates a very strong effect of age on the human bacterial microbiota of the NP. Interestingly, children at a very young age seemed to display a significantly lower dispersion as compared to older children (unweighted distances from the centroid; Tukey’s HSD test; *P* < 0.001; Figure 3D), indicating that an increase in age leads to a more heterogeneous microbial community structure. In contrast, all comparisons of weighted distances from the centroid were non-significant (Figure 3B), suggesting a stronger effect of community structure compared to community composition on multivariate dispersion across groups.

### Microbiota of NP and MEF Show Highest Similarity When *S. pyogenes*, *H. influenzae* or *S. pneumoniae* Are the Predominant OTUs

**FIGURE 4:**
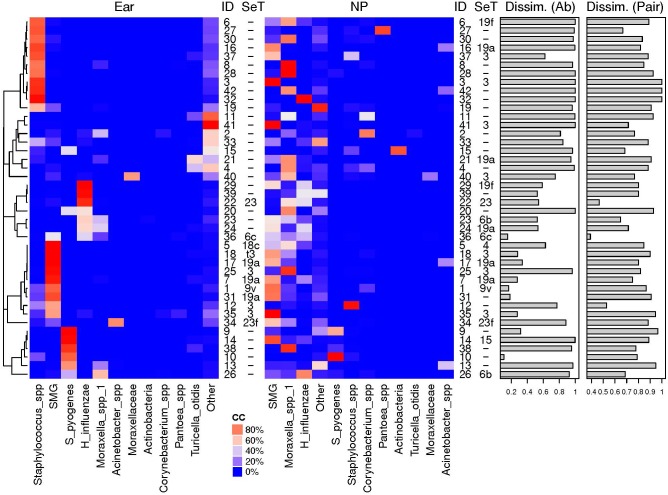
Relative abundances of most abundant OTUs in MEF from ear and NP. Shown are the heat maps of the relative abundances of the most abundant OTUs in % [illustrated by a distinct color code (CC)]. The pneumococcal serotpyes (SeT) are indicated. Dissimilarly values based on abundance based Ružička (Dissim. Ab) and presence-absence based Jaccard (Dissim. Pair) distance values between the two different sample types of the same individual (ID) are shown as bar plots. High similarity of the bacterial microbiota of NPS and MEF when *S. pyogenes*, *H. influenzae* or *S. pneumoniae* were the predominant OTUs and overall high negative predictive values for NPS based prediction of MEF (see Table 2 and text for more details). SMG, *Streptococcus mitis* group members (including *Streptococcus pneumoniae*).

We then compared the NPS microbiota profiles with the corresponding MEF profiles from 42 children. The 11 most abundant OTUs are illustrated (Figure 4) while all others were grouped (“others”). High dissimilarity values between the microbiota profiles of the NPS and the MEF were received if *Staphylococcus* spp. and “others” were the predominate OTUs within the MEF. Increased similarity was seen if the predominate OTUs within the MEF were *S. pyogenes*, *Streptococcus mitis* group (includes *S. pneumoniae)* and *H. influenzae*.

**FIGURE 5:**
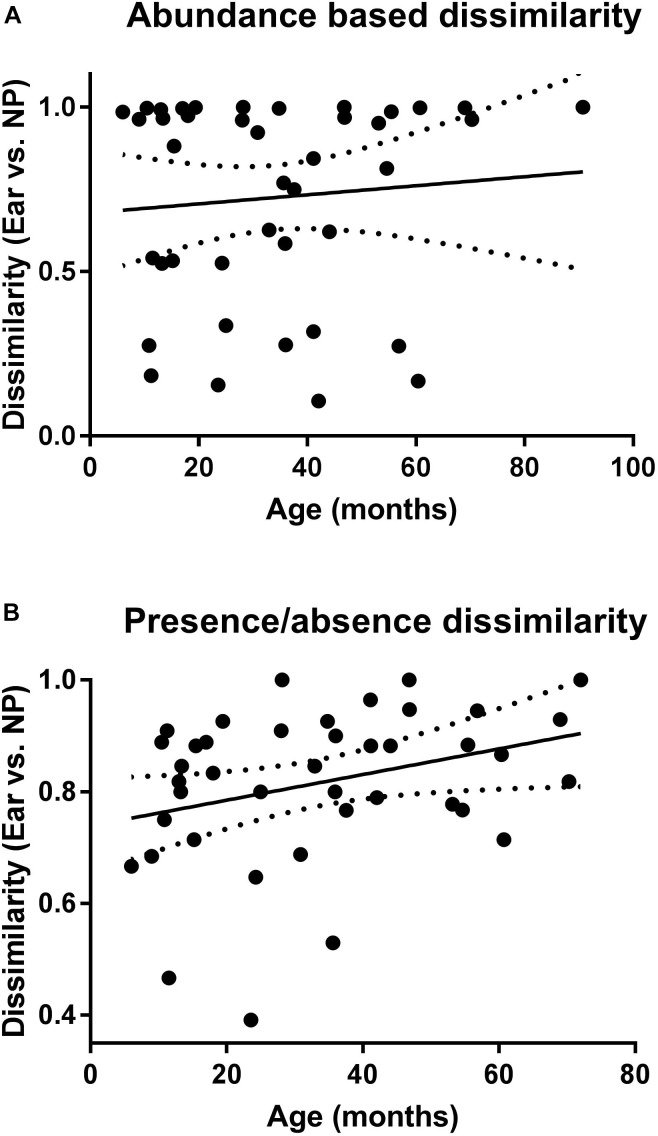
Correlation of the microbiota of NP and MEF according to age. Shown are the abundance **(A)** and presence/absence **(B)** based dissimilarity values (for NP and MEF) for the children with AOM at different ages. Linear regression lines are indicated. *P*-value is significant for the presence/absence based dissimilarities (*P* = 0.04).

Calculation of the correlation between dissimilarity values and age of the children with AOM (Figure 5A,B) revealed a significant positive correlation for the presence/absence-based dissimilarity values (Figure 5B), which was not the case for the abundance-based values (Figure 5A).

Finally, we determined the sensitivity/specificity of individual OTUs and the pneumococcal serotypes for the prediction of corresponding MEF results (Table 2). Highest sensitivity/specificity values were achieved for *S. pyogenes* and the pneumococcal serotypes. For one patient, the serotyping results were discordant which might be due to multiple colonization of different *S. pneumoniae* (Brugger et al., 2009, 2010). Concerning the other OTUs, some were more often found in the MEF (especially *Turicella* spp.) while the opposite was true for e.g., *Corynebacterium* spp., *Moraxella* spp. and *H. influenzae*. In general, positive predictive values (PPV) revealed a considerable, pathogen-dependent variability while the negative predictive values (NPV) were found to be consistently high for most of the OTUs (Table 2).

**Table 2:** Sensitivity and specificity MEF/NP based on pneumococcal culture and presence and absence of the eight most frequent OTUs in the NPS and ears, respectively.

	NP+MEF+	NP–MEF–	NP–MEF+	NP+MEF–	Sensitivity (95% CI)	Specificity (95% CI)	PPV, % (95% CI)	NPV, % (95% CI)
Pneumococcal culture	10^∗^	18	2	12	83 (52 – 98)	60 (41 – 77)	45 (33 – 58)	90 (71 – 97)
OTU name								
SMG	23	3	2	14	92 (74 – 99)	18 (4 – 43)	62 (56 – 68)	60 (22 – 89)
*Moraxella* spp.	10	9	1	22	91 (59 – 100)	29.0 (14 – 48)	31 (25 – 38)	90 (56 – 98)
*H. influenzae*	8	18	3	13	73 (40 – 94)	58.1 (39 – 75)	38 (26 – 52)	86 (69 – 94)
*Staphylococcus* spp.	10	13	14	5	42 (22 – 63)	72.2 (47 – 90)	67 (45 – 83)	48 (37 – 59)
*S. pyogenes*	6	33	2	1	75 (35 – 97)	97 (85 – 100)	86 (46 – 98)	94 (83 – 98)
*Corynebacterium* spp.	2	28	1	11	67 (9 – 99)	72 (55 – 85)	15 (7 – 32)	97 (85 – 99)
*Acinetobacter* spp.	1	36	1	4	50 (1 – 99)	90 (76 – 97)	20 (5 – 57)	97 (90 – 99)
*Turicella otitidis*	0	31	10	1	0 (0 – 31)	97 (84 – 100)	0	76 (74 – 77)


*^∗^Serotyping revealed that in 9 of 10 isolates, the serotype was identical in the middle ear fluid (MEF) as compared to the nasopharynx (NP). SMG, *Streptococcus mitis* group; OTU, operational taxonomic unit; PPV, positive predictive value; NPV, negative predictive value; CI, confidence interval.*

## Discussion

This study analyzed the bacterial microbiota profiles from 286 children with AOM (286 NPS and 42 MEF). In the NP, we noted an increase in bacterial richness and beta dispersion with age and a decrease in evenness (Figure 2, 3). This indicates the development of a more distinct bacterial microbiota profile toward the end of the sixth year of life. In addition, we found high similarity of the bacterial microbiota of NPS with MEF if the predominant OTU within the MEF was *S. pyogenes*, *H. influenzae*, or *S. pneumoniae*. The opposite was true for *Staphylococcus* spp. and others (Figure 4 and Table 2).

We performed 454 sequencing in our study, a technique which has been replaced by more high-throughput platforms, e.g., Illumina. However, the nasal microbiota has a rather simple, low-diversity profile (Escapa et al., 2018) and in order to achieve an approximate OTU coverage of 70% (which is mainly recommended) there is no need for a large amount of sequencing reads (Hilty et al., 2012). In addition, 454 sequencing produced longer reads (as compared to Illumina) which increased the resolution for OTUs. Most importantly, our protocols have been systematically investigated as we have previously done validation work on mock communities using a similar design (Brugger et al., 2012). This included using different primers [i.e., 8F (for V1–V5) versus 341 F (for V3–V5)] and investigating the detection limit of bacteria (i.e., mock communities) which are very well known to be present in the NP of children. Using the primer 8F, the detection limit was about 30 genome copies μl^-1^ with the exception of *H. influenzae* and *M. catarrhalis* (100 copies μl^-1^). As for the observed difference of the detection limit, this is probably due to a SNP present in the conserved region of the forward primer 8F and therefore suboptimal PCR efficiency. There are no SNPs in the primer regions of 341F and 907R for the bacteria mentioned above and we therefore concluded this primer pair being appropriate for the study of the bacterial microbiota of the NP. Using Illumina MiSeq, the same primer design cannot be used as the reads are not long enough.

There is a lack of studies like ours describing the nasopharyngeal bacterial microbiota in children with AOM up to 7 years of age. However, data exists for infants at a younger age describing a distinct profile for the first 3 months of life with increased relative abundances of Staphylococcaceae and Corynebacteriaceae (Laufer et al., 2011; Biesbroek et al., 2014a,b; Mika et al., 2015; Teo et al., 2015; Bomar et al., 2017; Salter et al., 2017). In our study we found an increase of Staphylococcaceae and Corynebacteriaceae in children older than 3 years. This may indicate the transition toward a more “adult”-like composition of the microbiota as these two families are very often identified within the adult microbiome (Frank et al., 2010; Camarinha-Silva et al., 2014; Bomar et al., 2017). This transition may take place due to a more mature immune response resulting in less episodes of AOM in older children. In addition, common to our and other studies using molecular or conventional culture methods are the high (relative) abundances of Moraxellaceae and Streptococcaceae within the first 2 years of life (Faden et al., 1994, 1997; Bogaert et al., 2004, 2011; Hilty et al., 2012). However, in our study, we show that the abundance of Moraxellaceae drastically decreases in children aged more than 2 years.

A further major finding of our study was the discovery of a significant increasing correlation of age and dissimilarly of the microbiota of NP as compared to the MEF. Based on bacterial cultures, a recent study has observed that *S. pneumoniae* and non-typeable *Haemophilus influenzae* nasopharyngeal colonization increases significantly between 6 and 30–36 months of age, but as children get older the relationship between potential otopathogen in the NP with detection in MEF is weakened (Kaur et al., 2014). Similarly, a high prevalence of nasopharyngeal colonization but low frequency of *S. pneumoniae* as an etiology of AOM with age, especially after children became >18 months of age has been reported (Syrjanen et al., 2005). Our bacterial microbiota-based analyses showed similar findings as compared to culture-based studies. However, NP microbiota patterns from children below 2 years of age are still not very good surrogates of MEF cultures in children as dissimilarity values were quite high.

A recent systematic review of the literature reported the concordance between culture test results of the NP and MEF samples for the most prevalent microorganisms in children with otitis media (van Dongen et al., 2013). Culture-based AOM studies revealed higher PPVs (for *H. influenzae* and *S. aureus*) and lower PPVs (for *M. catarrhalis*) as compared to our study, respectively (van Dongen et al., 2013). As for *S. aureus*, this might be in part due to the fact that all MEF in this study were derived from cases with spontaneous tympanic membrane perforation which have been reported to contain a higher proportion of *S. aureus* (Brook and Gober, 2009).

However, with the exception of *S. aureus*, we received much higher NPVs as compared to culture-based studies. Our NPVs were also higher if compared to a study which was using a series of polymerase chain reactions in order to detect bacteria (Yatsyshina et al., 2016). This means that high-throughput sequencing of the bacterial microbiota of the NP results in decent NPVs for finding the pathogens within the MEF while this is not the case for the PPVs (with the exception of *S. pyogenes*).

Recently, Man et al. (2018) compared the microbiota of NP and MEF in almost 100 Dutch children under 5 years of age with AOM in children with tympanostomy tubes and also reported a high overall NPV (on OTU level) suggesting that the NP might be the main source of MEF bacteria in otitis media. The overall PPV was 0.4 (95%CI 0.39 – 0.42) which is in line with our results. However, significant quantitative correlations between NP and tympanostomy tube otorrhea (TTO) samples for *Haemophilus influenzae*, *S. aureus*, and *P. aeruginosa* abundance were reported (Man et al., 2018). In agreement with our results, *T. otitis* was associated with MEF supporting its role as an otopathogen (Man et al., 2018).

This study has some major strengths. First, we have included a large number of NPS (*n* = 286) from children with AOM at different ages (up to <7 years of age). Patients were recruited from different outpatient settings from a well-defined sentinel network and medical diagnosis was done based on CDC case definition. In addition, we have included 42 paired samples of NPS and MEF allowing the investigation of the appropriateness of the NP as a proxy for the microbiota of the MEF. Apart from 16S rRNA sequencing, we also performed culturing for *S. pneumoniae* and subsequent serotyping for all positive samples.

This study has also some major limitations. Within our study, we did not investigate homogeneity of nasopharyngeal microbiota at the different locations of the nose of children with AOM. However, results from a recent study suggest that the microbiota at the different locations of the nose of children with AOM is almost homogeneous, irrespective of the clinical signs (Kaieda et al., 2005). We also only analyzed the bacterial microbiota and did not include virus detection in this study (e.g., by shotgun metagenomics sequencing). In addition, MEF collection was biased toward aggressive forms of AOM as only material of spontaneous perforations was collected which resulted in only 48 NP/MEF paired analyses. Also, based on the design of the surveillance program the information about the exact duration of the AOM episode as well as the time of tympanic membrane perforation was not recorded. Furthermore, sample acquisition was not standardized (i.e., no tympanocentesis samples) which could have resulted in contamination with skin flora. Thus, we cannot rule out that otitis externa might have been diagnosed as AOM in some cases (this could be reflected by the higher relative abundance of Staphylococcaceae in MEF compared to NPS, however, the pneumococcal isolation from NPS highly matched with pneumococcal isolation in MEF).

Further studies investigating the development of the bacterial microbiome including AOM should follow a prospective design (using a power calculation based on our results) to reduce the risk of unmeasured confounders.

## Conclusion

In conclusion, this study revealed varying nasopharyngeal bacterial microbiota patterns according to age and moderate to high NPVs for the prediction of MEF OTUs based on analyses from the NP in a subset of children with spontaneous tympanic membrane rupture where we simultaneously characterized the bacterial microbiota of NPS and MEF. The NP of children with AOM serves as a moderate and insufficient proxy for the bacterial communities of the MEF at a very young and more advanced age, respectively. Given these results as well as previous culture (or species-specific PCR) based studies, the nasopharyngeal bacterial microbiota does not necessarily reflect the one of the middle ear in AOM and should only be used with caution for clinical decision decision-making. This might be in part due to the complex disease pathology in AOM, which also involves viruses and biofilms.

## Data Availability

The datasets generated for this study can be found in National Center for Biotechnology Information Sequence Read Archive, SRR077426, SRR086165, SRR086166, and PRJEB23228.

## Ethics Statement

The overall AOM surveillance program has been part of the governmental public health surveillance and has therefore been exempted from approval by Institutional Review Boards and the need for written consent from parents. Informed oral consent was received from the parents.

## Author Contributions

SB and MH were investigators in this study and contributed to the study design. SB, JK, WQ, LB, AO, and MH contributed to data collection and analysis. SB, JK, and MH contributed to data interpretation. All authors contributed to the writing, review of the report, and approved the final version of the manuscript.

## Conflict of Interest Statement

MH received an educational grant from Pfizer AG for partial support of this project. However, Pfizer AG had no role in the data analysis and content of the manuscript. The remaining authors declare that the research was conducted in the absence of any commercial or financial relationships that could be construed as a potential conflict of interest.

## Footnotes

1 http://www.R-project.org

## Abbreviations

CI: confidence interval
MEF: middle ear fluid
MID: multiplex identifier
nMDS: non-metric multidimensional scaling
NP: nasopharynx
NPS: nasopharyngeal swab
OTU: operational taxonomic unit
PCV: pneumococcal conjugate vaccine
rRNA: ribosomal RNA
SD: standard deviation
SDI: shannon diversity index
