# Supplementary material for: Age-Dependent Dissimilarity of the Nasopharyngeal and Middle Ear Microbiota in Children With Acute Otitis Media
Source: Front Genet. 2019 Jun 19;10:555. doi: 10.3389/fgene.2019.00555 (PMC6593076; doi:10.3389/fgene.2019.00555)

Supplementary Figure 1; Richness (S) and Chao for nasopharyngeal swabs

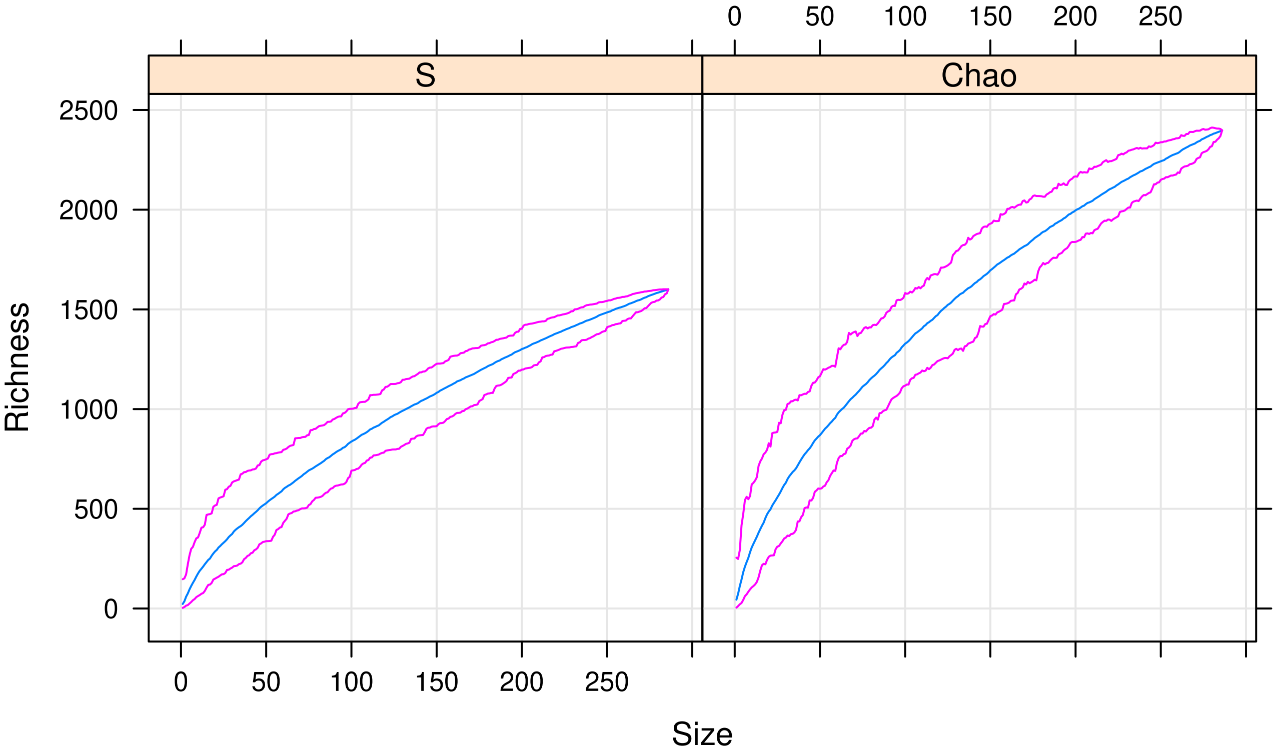

Supplementary Figure 2; Richness (S) and Chao of middle ear fluid samples

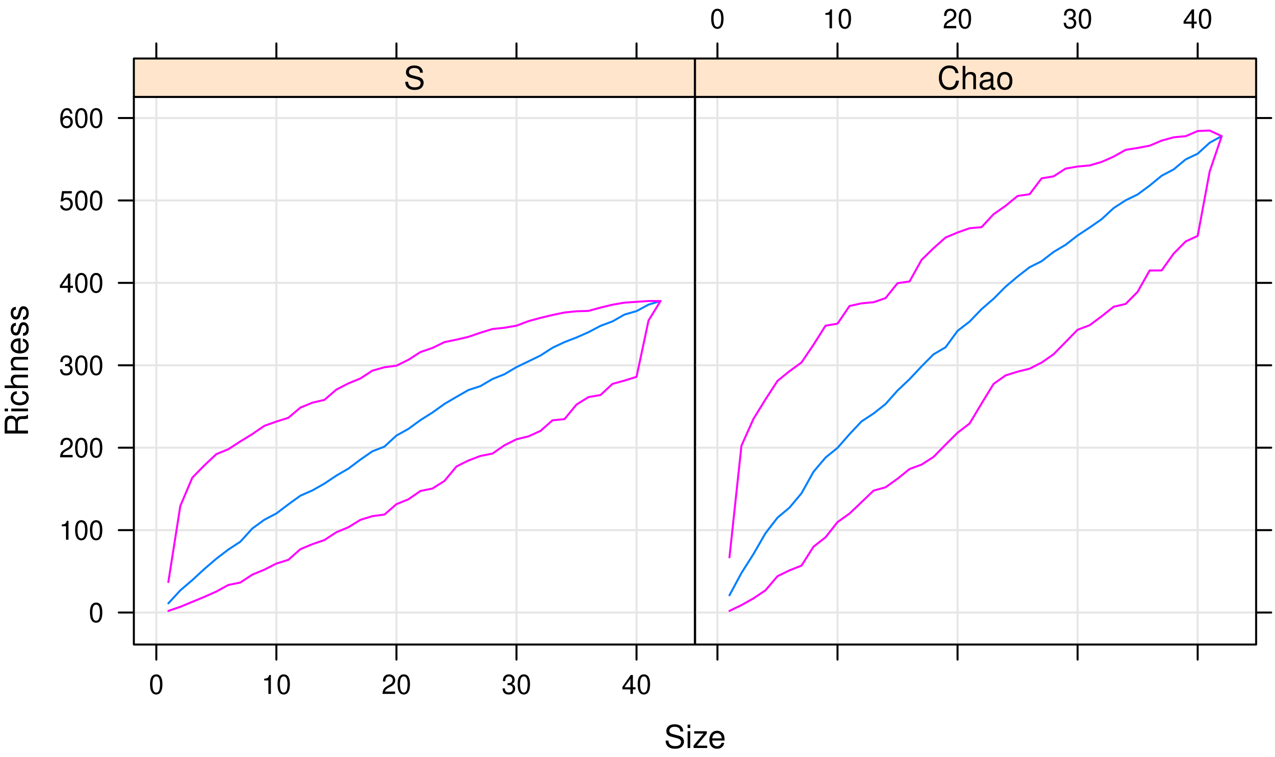

Supplement: Supplementary file 1 [file Data_Sheet_1.pdf]
